# A sui generis QA approach using RoBERTa for adverse drug event identification

**DOI:** 10.1186/s12859-021-04249-7

**Published:** 2021-10-21

**Authors:** Harshit Jain, Nishant Raj, Suyash Mishra

**Affiliations:** 1 ZS Associates, Bengaluru, India; 2 University of Massachusetts Amherst, Amherst, USA; 3 ZS Associates, London, UK

**Keywords:** Adverse drug event, RoBERTa, Question-answering, Entity-relation extraction, Healthcare

## Abstract

**Background:**

Extraction of adverse drug events from biomedical literature and other textual data is an important component to monitor drug-safety and this has attracted attention of many researchers in healthcare. Existing works are more pivoted around entity-relation extraction using bidirectional long short term memory networks (Bi-LSTM) which does not attain the best feature representations.

**Results:**

In this paper, we introduce a question answering framework that exploits the robustness, masking and dynamic attention capabilities of RoBERTa by a technique of domain adaptation and attempt to overcome the aforementioned limitations. With formulation of an end-to-end pipeline, our model outperforms the prior work by 9.53% F1-Score.

**Conclusion:**

An end-to-end pipeline that leverages state of the art transformer architecture in conjunction with QA approach can bolster the performances of entity-relation extraction tasks in the biomedical domain. In particular, we believe our research would be helpful in identification of potential adverse drug reactions in mono as well as combination therapy related textual data.

## Background

Recent advancements in drug development and approval have bolstered our healthcare ecosystem. However, this rapid paced development is accompanied by an increase in associated risks. Adverse drug events (ADEs) form an integral component of those risks. An ADE is defined as “an injury resulting from a medical intervention related to a drug” [1].

These events create an economic burden over the system. A study by Rocchiccioli et al. showed “a statistically significant increase in all direct costs (inpatient,outpatient, therapy) during post-ADE period (+US$1310 for all ADEs and +US$1983 for preventable ADEs, versus the pre-event period)” [2–4]. Further, national estimates are indicative of the fact that ADEs contribute at least an additional US$ 30 billion to US healthcare costs [5, 6]. Thus, it becomes an utmost requirement to identify these ADEs at an early stage from biomedical literatures, EHR data and other sources in order to avoid additional costs incurred in patient management while improving pharmacovigilence practices at the same time.

Several adverse drug events have been reported to U.S. Food & Drug Administration (FDA) through Federal Adverse Event Reporting System (FAERS). These reports submissions are voluntary and hence they may suffer from cases of massive under-reporting [7]. Hence, researchers have started moving towards more automated approaches in machine learning. There has been a gradual shift towards using natural language processing (NLP) based methods. Early attempts have incorporated the use of resources like NLM’s MetaMap, Unified Medical Language System (UMLS) etc. [8] to extract drugs for ADE identification tasks. However, a major limitation of these approaches is that they are not able to capture the causal relationships between drug and ADE properly.

Another popular approach in the community has been to treat this problem as entity recognition and relation identification task. This approach has further been tackled using either a *1. pipeline method* where entity recognition tasks are done first followed by relation identification or *2. a joint training method* where training weights are shared between these two sub-tasks so that errors don’t accumulate [9–11].

Miwa and Bansal [9] proposed an end-to-end joint relation extraction model where they stacked bidirectional tree-structured LSTMs on bidirectional sequential LSTMs. Their work inspired many researchers to take this knowledge to biomedical entity and relation extraction tasks.

Li et. al [10] proposed a neural joint model for ADE relation extraction from biomedical texts. They utilized Bi-LSTM architecture for biomedical entity recognition using concatenation of character representation, POS embedding and word embedding as input features. This layer shared partial parameters with another Bi-LSTM layer which was tuned during a joint training process. These works using Bi-LSTMs helped to make a significant progress in entity-relation extraction tasks. However, the multi-head self attention capabilities allowed transformers to capture long range dependencies efficiently. This along with contextual dense representations from pre-training using a very large corpus allowed a better feature extraction capability.

Most of these works have utilized different variants of long short term memory (LSTM) networks for both entity recognition and relation identification tasks. Though these networks have shown promising results, they fail to capture best feature representations.

There have been ground-breaking developments in NLP with the advent of transformer architectures like BERT [12], GPT [13], RoBERTa [14] etc. Transformer networks utilize the power of multi-head self-attention mechanism to capture context-sensitive embeddings and interactions between tokens. Since they have been pre-trained on extremely large documents for language modeling task, they have shown state of the art results with downstream tasks like sentiment analysis, question answering, machine translation etc.

Li et. al [11] in their work formalize a QA framework using BERT architecture. Their research emphasizes how question based queries can encode the important information for entity-relation class identification in the question and at the same time provide a natural way of jointly modeling entity and relation. Eberts and Ulges [15] present a span-based joint entity and relation extraction model with transformer pre-training. They add a span classification layer that filters entities from non-entities. The filtered entities are then used for relation identification purposes.

We build upon the learnings of these research endeavours to devise a new QA framework for ADE identification task using a more powerful transformer architecture RoBERTa that we describe in the next section.

Rest of our work is organized as follows. We first discuss our approach in detail i.e. system architecture, dataset, experimental setup, training and evaluation metrics. This is followed by a discussion of the experimental results and in the end we make a conclusion.

## Method

**Fig. 1:**
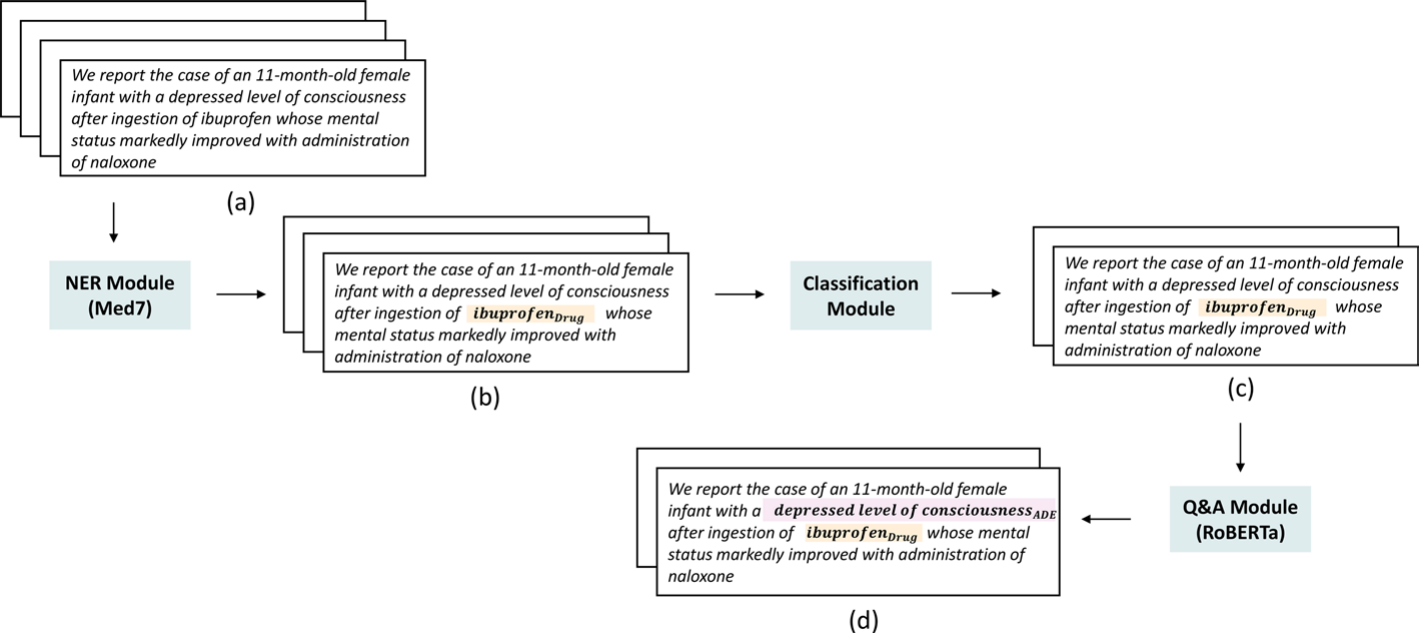
End-to-end system architecture. (**a**) Positive (ADE) and Negative (Non-ADE) sentences are passed through NER module (**b**) NER module filters sentences which don’t have drug entity mentions (**c**) Selected sentences at NER stage are passed through Classification Module which filters out sentences where Drug-ADE relation is less probable (**d**) RoBERTa QA Module identifies ADE relationship (e.g. depressed level of consciousness) corresponding to the selected drug entity (e.g. ibuprofen)

In this section, we introduce our system architecture (Fig. 1) and explain three different modules it invokes in a sequential manner.

### Entity recognition module

Name entity recognition has been identified as a pivotal task in NLP. Classification of words from biomedical text into predefined categories like drug, disease, dosage etc. is a challenging problem. Many researchers have faced an issue of unavailability of annotated medical corpus due to which model generalization becomes difficult.

In our system, to identify the drug names in a given phrase, we leveraged recently developed Med7 [16] NER module which is trained on a collection of 2 million free-text patients’ record from MIMIC-III corpus followed by fine-tuning on the NER task. *“It has attained a lenient micro-average F1 of 0.957 across seven different categories (Dosage, Drug, Duration, Form, Frequency, Route, Strength)*”.

Few output samples from NER Module for drug identification can be found below *(highlighted text in blue indicates predicted drug entities)*:

*a. we report the case of a patient with multiple myeloma who developed acute life-threatening water intoxication following treatment with oral* *and low dose* *intravenous* 

*b. we report the case of a 58 year old patient who, after 2 days of treatment with* * and*  , *manifested acute pancreatitis*

*c. a 26-year-old japanese man, who had been receiving medical attention for ulcerative colitis for one year, presented with diffuse erythema and pustules on his face and trunk, malaise, and fever up to 39 degrees c one day after the administration of*  .

*d. we report the results of three cases of limited-stage SCEC treated with combination therapy using* *(CBDCA) and* *(VP-16) and radiotherapy.*

### Classification module

Denoising and extraction of relevant information in textual data is a crucial step as it improves the learning mechanism and generalizing capability of any model. After recognizing the drug entities in entity recognition module,in order to identify the phrases where at least one drug and adverse event pair coexists at stage 2, we trained a Bi-LSTM [17] based binary classifier on ADE sentences and cross-validated it in K-Fold setting.

This model aims to filter out phrases with no presence of drug and adverse event pair and helps in improving the performance of our Q&A module and thereby making it more robust.

Out of the aforementioned samples in the Entity Recognition Module, classifier module rejects sample (d) as it does not contain an adverse event corresponding to the identified drug i.e. *etoposide* and passes sample (a), (b) and (c) to the Q&A Module.

### Q&A module

Given a passage of text with a user query, a question-answering system discovers a span of text in the passage that best describes answer to the question being asked.

BERT [12] based pre-trained language models have achieved state-of-the-art performances on multiple tasks like semantic role labeling, question answering, machine translation, etc as it has been trained on large scale corpora and generalizes the downstream task very well. It can learn the extremely complex representation in text with a transformer-based [18] self-attention mechanism and play a crucial role in improving varieties of NLP systems.

Liu et. al [14] in their paper that introduces RoBERTa, observed BERT to be “*significantly undertrained*” and also found out that with a better selection of hyperparamerters and training size, its performance could be considerably improved. RoBERTa, a re-implemented version of BERT in FAIRSEQ [19], has been trained with different learning rate, number of warm-up steps, batch size and out-performs state-of-the-art results on GLUE, RACE and SQuAD dataset.

**Fig. 2:**
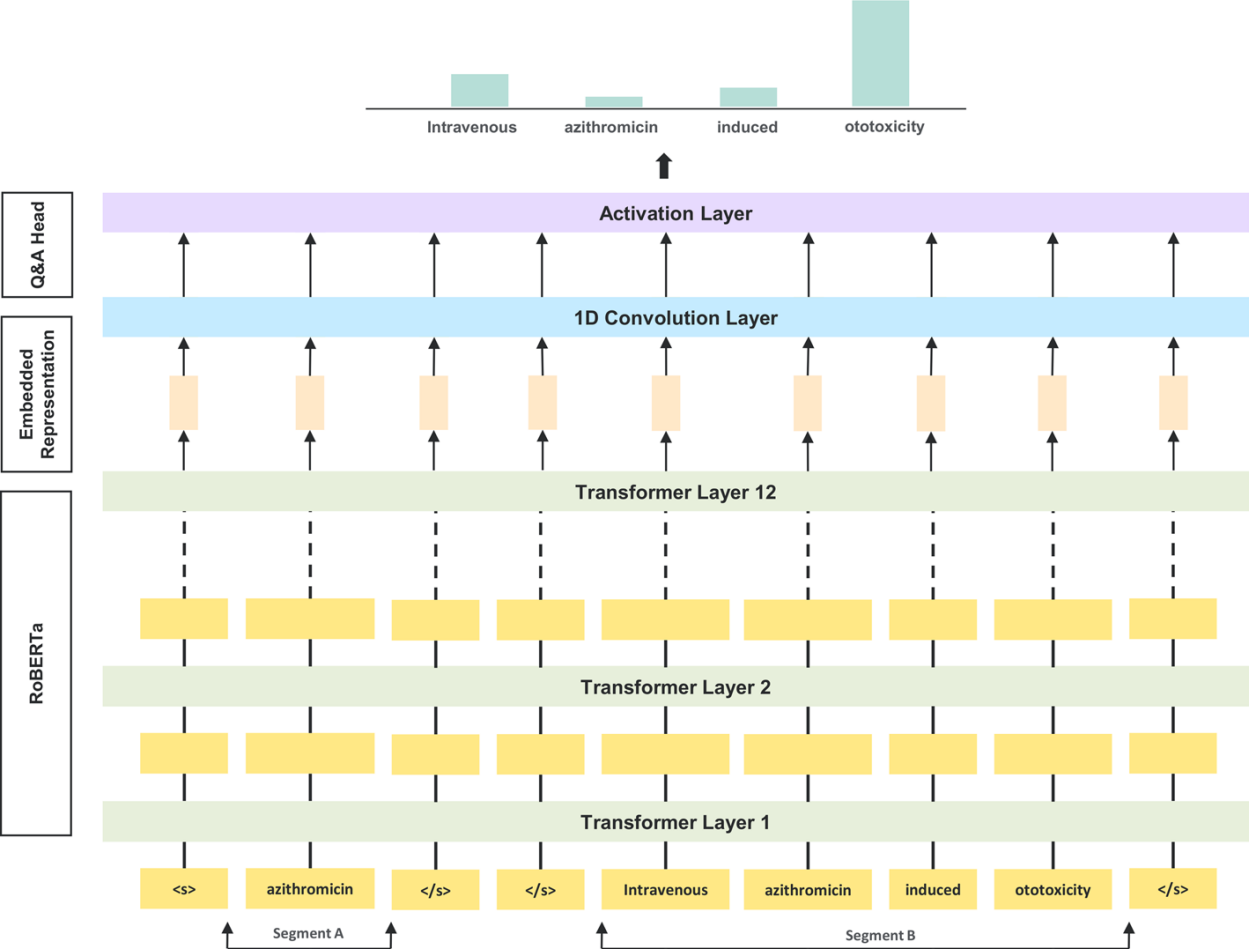
Q&A Module for Adverse Drug Event Identification. Input segment A consists of a drug acting as an alias for question and segment B consists of context where an ADE is mentioned. Encoded representation of segments passed as an input through 12-layered transformer network. 1-D convolutional layer is applied on top of 768 dimensional embedded representations followed by a softmax activation to identify the ADE

We use RoBERTa base model and fine-tune it on drug-related adverse effects corpus to identify the adverse event corresponding to a drug. To perform fine-tuning, we utilize pre-trained weights of RoBERTa’s 12 layer transformer network and add a CNN based Q&A head (Fig. 2) on top of it. First we process the data in desired input format for RoBERTa into 2 segments A and B. Segment A consists an encoded vector of drug treated as a question followed by segment B that consists another encoded vector of context/sentence where adverse event is mentioned. Then we pass this processed data into a 12-layered transformer network of RoBERTa and use its output that represents the 768 dimensional learnt embeddings of encoded input for further processing.

After this, we apply a one dimensional CNN layer with a (1 x 1) convolution filter that creates a feature map of these embeddings followed by a flatten and a softmax activation layer to predict the probability of start/end tokens of the adverse event present in a span of the given text. Since 1D CNN layer renders the network with property of being “translationally invariant”, a pattern of adverse event learnt at one position in a sentence can be easily identified at a different position as the same input transformation is applied on every patch.

Output samples from Q&A Module for the previous examples after passing from classification module can be found below *(highlighted text in red indicates predicted ADE and the one highlighted in blue indicates predicted drug)*:

*a. we report the case of a patient with multiple myeloma who developed acute life-threatening* *following* *treatment with oral indomethacin and low dose intravenous*  (Actual Drug: cyclophosphamide, Actual ADE: life-threatening water intoxication)

*b. we report the case of a 58 year old patient who, after 2 days of treatment with roxithromycin and*  , *manifested*  (Actual Drug: betamethasone, Actual ADE: acute pancreatitis)

*c. a 26-year-old japanese man, who had been receiving medical attention for ulcerative colitis for one year, presented with* *up to 39 degrees c one day after the administration of*  (Actual Drug: salazosulfapyridine, Actual ADE: diffuse erythema and pustules on his face and trunk, malaise, and fever up to 39 degrees c)

There could be scenarios where a single drug could be associated with multiple ADEs and vice-versa. In cases where the NER Module identifies multiple drugs in a given sentence, these drugs would serve as separate questions for the QA Module in the pipeline. In the aforementioned examples (a) and (b), the NER module identifies two different drugs. These drugs act as separate questions in the QA Module where the multiheaded self-attention mechanism in RoBERTa produce same outcome i.e.  for case (a) and  for case (b). But for the ease of understanding, we have demonstrated this with only one drug out of the two in the above examples.

On the flip side, in samples where multiple ADEs are present corresponding to a given drug, Q&A module aims to identify a span of text which has the highest matching probability corresponding to the question posed. There could be cases where the QA module might miss some part of the span and in those scenarios, it would imply that the probability of that unidentified span was not high enough to be considered. In example (c) mentioned above, Q&A module is able to identify  but misses the ending part of the span i.e.  .

### Dataset and evaluation metrics

We use drug-related adverse effects corpus [20] containing sentences from 1644 PubMed abstracts. These sentences are divided into 2 categories (i) ADE (ii) Non-ADE (Table 1). Former consists of sentences where at least one pair of drug and its adverse effect is present while latter consists of sentences with no such pair.

**Table 1:** Distribution of sentences in dataset

Category	Number of unique sentences
ADE	6617
Non ADE	16688

Examples for ADE and Non-ADE instances are:

- **ADE:** *14-year-old girl with newly diagnosed sle developed a* ***pruritic bullous eruption**** while on* ***prednisone***
- **Non-ADE:** *This patient did not have any predisposing factors for the development of an aortic thrombus before the chemotherapy was initiated.*

To gauge the performance of our system, we use common performance metrics such as Precision, Recall and F1.

1 $$\begin{aligned} P&= \frac{TP}{TP+FP}, \nonumber \\ R&= \frac{TP}{TP+FN}, \nonumber \\ F1&= \frac{2*P*R}{P+R} \end{aligned}$$

### Training

We perform training for 2 different modules involved in our system i.e. Classification and Q&A.

(i) For classification module, we construct a train and test dataset by selecting randomly sampled 9931 training and 1272 testing instances. We train a Bi-LSTM with Adam optimizer [21] minimizing binary-crossentropy loss in stratified K-Fold (k=10) setting to ensure consistency in our model performance. For final prediction on hold out dataset, we use an ensemble of these 10 Bi-LSTMs. The distribution of target variable in train and test sets can be found in Table 2.

**Table 2:** Distribution of target variable in train and test for Classification Module

Positive (ADE)	Negative (Non-ADE)	Dataset
3976	5955	Train
610	662	Test

(ii) To predict ADE corresponding to a drug, we create the train & and test data by determining 5955 training and 662 testing instances from ADE sentences with random sampling & perform fine-tuning on these 5955 sentences.

We train the entire architecture for Q&A module in K-Fold setting (k = 5) with 3 epochs each on 12GB Nvidia P100 GPU. We use an ensemble of prediction probabilities for start & end tokens generated by models trained on each of the 5 folds. We set a learning rate of 3e-5 for Adam optimizer and categorical-crossentropy as our loss function with a label smoothing of 0.1.

## Results

Our RoBERTa based QA approach utilizes *drug* entity passed as a *question* to determine *answer* i.e. *adverse drug event* in the given context. We determine the performance at two levels: (i) Performance of individual modules (ii) Performance of entire architecture (system).

Previous works [10, 11, 15] in the field focus on approaches that leverage either a joint training approach or a cascading pipeline approach to identify entities and then classify those extracted entities to ascertain existence of any relationship. However, our approach as described in previous section is not tailored similar to these settings. Hence, a direct comparison of individual modules is not feasible. We elucidate our approach for calculation of system’s performance using these three modules together.

### Performance of individual modules

Table 3 details the performance of Classification and QA module corresponding to their selected training and validation set as described in the previous section. The results for classification module are reported based on mean results from 10-Fold validation sets. The presence of Non-ADE sentences where drug is either not known or wrongly identified is expected to create a bias in understanding of effectiveness of RoBERTa QA module for ADE identification tasks. Hence, we use only ADE sentences where drug is known beforehand for determining the performance of Roberta QA framework. In this scenario, the precision would equal to 1 and hence we use recall to gauge the true efficacy of QA module.

**Table 3:** Performance evaluation of classification & QA Modules

Metrics	Classification module	QA module
Precision	82.74	–
Recall	81.44	87.37
F1	82.06	–

### Performance of end-to-end architecture

Errors generated by different components in a system create a cascading effect and this aggregation of errors might render the system to a practically futile state.

**Fig. 3:**
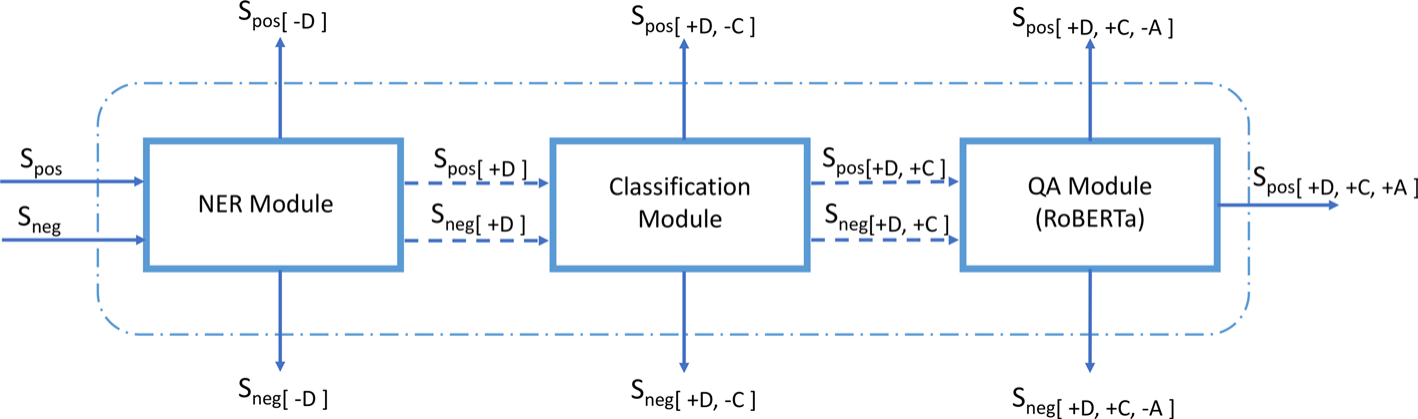
End-to-End System Architecture’s Overall Performance Calculation. Sentences in two sets (Spos & Sneg) are passed into the system with an objective to identify drug-ADE relationship from Spos and removal of all the Sneg instances. Classification matrix generated at each stage is leveraged for final calculation of precision, recall and F1 metrics

In real-world applications, the overall task comprises of drug identification, noise removal and then drug-ADE relationship identification. In that process, obliteration of noisy textual information should also be accounted into the success criteria for smooth functioning of a NLP pipeline. We describe our approach to calculate the efficacy of entire system through Fig. 3.

At each stage either a sentence is eliminated from system or it moves to next module for operation. S_pos_ [+D,+C,+A] denotes that among the positive sentences passed as input, these sentences had drug and were sent to QA module by classification module where ADE was accurately identified by RoBERTa QA architecture. S_neg_[-D], S_neg_[+D,-C] contribute to correctly removed instances from system. Similar analogy for their S_pos_ counterparts identifies misclassified samples from the system. Equations for calculation of Precision, Recall and F1 scores for the entire architecture can be calculated using (1) and the modified definitions below:

2 $$\begin{aligned} TP&= S_{neg[ -D ]}+S_{neg[ +D, -C ]}+S_{pos[ +D, +C, +A ]} \nonumber \\ FN&= S_{pos[ -D ]}+S_{pos[ +D, -C ]}\nonumber \\ FP&= S_{pos[ +D, +C, -A ]}+S_{neg[ +D, +C, -A ]} \end{aligned}$$

We detail the final performance metrics for the end-to-end system architecture in Table 4. We also visit the effectiveness of prominent approaches for ADE identification task in Table 5. After a thorough study of the relevant literature for ADE identification tasks, we observed that in joint training approaches, reporting of results is done in a way that the relation classification task also incorporates effects due to errors generated by entity recognition modules. Hence, we compare the results of relation classification task to the effectiveness of our entire end-to-end architecture.

**Table 4:** End-to-end system performance

P	R	F1
88.37	84.44	86.36

**Table 5:** Comparison with different methods

Methods	P	R	F1
CNN + Global features [22]	64.00	62.90	63.40
BiLSTM + SDP [10]	67.50	75.80	71.40
SpERT [15]	77.77	79.96	78.84
Our model (end-to-end)	88.37	84.44	86.36

With a closer and detailed inspection, we observe that even after error propagation effect, the overall effectiveness of our system is better than existing approaches for ADE identification.

## Discussion

The experimental results highlight the effectiveness of using an end-to-end pipeline comprising of NER, classification and RoBERTa based QA modules. Although our end-to-end architecture achieves promising results, we plan to improve on the NER component in the architecture by building a transformer based biomedical NER. Also, improvements might be achieved subject to further experimentation using BioBERT as they have been pre-trained on biomedical texts and might fit more aptly for this dataset. However, as we plan to extend this pipeline to social media platforms like twitter for pharmacovigilance based studies, the objective was to use a robust architecture where minimal use case based changes would be required.

## Conclusion

In this paper, we propose a novel approach for ADE identification tasks in biomedical texts. We use a classification module to denoise the information before we show how entity-relation extractions tasks in biomedical domain can be effectively modeled as a question-answering problem using transformers. Our end-to-end architecture achieves competitive performances with respect to the existing best systems for adverse drug event relation extraction tasks. We hope that our work would open up a new dimension in ADE identification and entity-relation extraction tasks in general.

## Data Availability

The dataset of ADE task can be downloaded at: https://sites.google.com/site/adecorpus.

## Abbreviations

QA: Question answering
Bi-LSTMs: Bidirectional long short-term memory networks
LSTMs: Long short-term memory networks
RoBERTA: Robustly optimized Bidirectional Encoder Representations from Transformers Approach
BERT: Bidirectional Encoder Representations from Transformers
GPT: Generative Pre-trained Transformer
UMLS: Unified Medical Language System
ADEs: Adverse drug events
FDA: Food & Drug Administration
FAERS: Federal Adverse Event Reporting System
NLP: Natural language processing
NER: Named Entity Recognition
GLUE: General Language Understanding Evaluation
RACE: ReAding Comprehension dataset from Examinations
SQuAD: Stanford Question Answering Dataset
CNN: Convolution neural network
